# Insecticidal activity of the linear pseudoscorpion venom peptide Ammogarypin revealed by functional profiling

**DOI:** 10.3389/fphar.2025.1661173

**Published:** 2025-09-24

**Authors:** Maurice Pierry, Ludwig Dersch, Jonas Krämer, Lennart Schulte, Kornelia Hardes, Tobias Kessel, Jens Grotmann, Maximilian Seip, Andreas Vilcinskas, Tim Lüddecke

**Affiliations:** ^1^ Animal Venomics Lab, Fraunhofer Institute for Molecular Biology and Applied Ecology, Giessen, Germany; ^2^ Institute for Insect Biotechnology, Justus Liebig University of Gießen, Heinrich-Buff-Ring, Giessen, Germany; ^3^ BMBF Junior Research Group “ASCRIBE”, Fraunhofer Institute for Molecular Biology and Applied Ecology, Giessen, Germany; ^4^ Department of Bioresources, Fraunhofer Institute for Molecular Biology and Applied Ecology, Giessen, Germany

**Keywords:** arachnida, venomics, biodiscovery, AMP, pest insects, biochemistry

## Abstract

Some pseudoscorpions produce venom to subdue prey and their venom components may be of translational interest in agronomy and beyond. However, only very few pseudoscorpion venom peptides have been functionally characterized as of yet. Here, we carry out a bioactivity profiling of Ammogarypin, a linear venom peptide recently identified in the venom of *Ammogarypus lawrencei*. We show, that the peptide causes signs of spastic paralysis and fatalities when injected in *Drosophila suzukii* flies and low toxicity when injected in *Myzus persicae* aphids, while no effects were recovered when fed to both insects. The toxin further has marginal effects on growth of *E. coli* and *S. aureus* bacteria and no effect on the viability of mammalian MDCKII cells and equine erythrocytes. While our assessment revealed low potential for agricultural translation of the peptide, our data shows that Ammogarypin appears to fulfill a function in prey capture. In contrast to other linear pseudoscorpion toxins, it seems to serve only a single biological function and does not defend the venom gland against microbial colonization, nor serving as spreading factor. This study adds to the growing body of literature revolving around the biodiscovery and biochemical ecology of pseudoscorpions, some of earths smallest and least understood venomous animal lineages.

## 1 Introduction

The global rise of insect pests and their inflicted economic damage is rooted in rapid adaptations and resistance to chemical synthetic insecticides (Pu and Chung, 2024; Bass et al., 2014). At the same time, there is a growing public concern about the negative impact on human health, the environment, and on biodiversity stemming from the use of chemical insecticides. This elicited recent efforts to restrict their use (Zhao et al., 2022; Chagnon et al., 2015; Zhang and Lu, 2022; Li et al., 2025). These limitations have sparked increasing interest in alternative, sustainable agricultural control measures against insect pests (Ragasruthi et al., 2024; Christiaens et al., 2020; Luna-Ramirez et al., 2017). In this framework, novel candidates for the development of agricultural leads with eco-friendly characteristics are urgently sought after (Christiaens et al., 2020; Luna-Ramirez et al., 2017).

Among the most promising sources of novel agricultural leads are venom peptides from arachnids, such as spiders, scorpions, and pseudoscorpions (Windley et al., 2012). Arachnids evolved chemically complex venoms that contain hundreds to thousands, primarily neurotoxic, peptides that facilitate prey capture, defence, and intraspecific competition (Lüddecke et al., 2022). Due to several hundred million years of selection, these components achieved an unprecedented degree of potency and target specificity, rendering them excellent leads for translational purposes (Windley et al., 2012; Pineda et al., 2014; Uzair et al., 2018). While the greatest potential of arachnid venom compounds is traditionally seen in biomedical applications, their evolutionary refinement for trophic purposes offers additional opportunities for translational research (Dresler et al., 2024; Windley et al., 2012; Pineda et al., 2014; Uzair et al., 2018). The vast majority of arachnids primarily feed on insect prey and hence their toxin arsenal is functionally optimized to act on molecular targets within the insect physiology (King and Hardy, 2013). Some arachnid toxins are known for their phyletic selectivity, i.e., their ability to target only specific insect lineages and often having little toxicity towards humans (King and Hardy, 2013). In addition, most arachnid toxins are peptides and as such biodegradable and easy to bioengineer towards increased potency (Dersch et al., 2024). Therefore, arachnid venom peptides have the potential to emerge as a prolific source of novel eco-friendly alternatives to existing pesticides and may be pivotal for a sustainable transition of the agronomy.

Among arachnid venom peptides, those of pseudoscorpions take an outstanding position. On one hand, pseudoscorpions represent the least studied group of venomous arachnids (Krämer et al., 2025). At time of writing only the venom of five species have been investigated (Krämer et al., 2021; Krämer et al., 2025; Santibáñez-López et al., 2018; Lebenzon et al., 2021; Santos and Coutinho-Netto, 2006), representing <0.2% of the venomous pseudoscorpion biodiversity (Hlebec et al., 2023). That said, some of the few so far described and functionally investigated toxins from this group displayed quite potent insecticidal activity in aphid feeding assays (Krämer et al., 2022). Therefore, they are believed to feature promising leads for future attempts to develop venom-derived insecticides targeting aphid, and potentially other, pests. However, despite the primary data suggesting a translational potential, little is known about the activity profile of pseudoscorpion toxins. The subsequent functional screening of hitherto unstudied components addresses a literature gap and more research is needed to understand how pseudoscorpion venom toxins function, what their biological role is, and how they could be translated into novel bioresources for agronomy and beyond.

Recently, a novel subset of structurally promising peptides has been identified in the venom of *Ammogarypus lawrencei*, yet their functional evaluation is pending (Krämer et al., 2025). One of these peptides is Ammogarypin. This peptide was originally described by us as Novel *Ammogarypus* Linear peptide 4 as it exhibits a high degree of sequence disparity from known venom peptides. Ammogarypin is a linear peptide exhibiting a notably long primary structure compared to previously discovered linear peptides from pseudoscorpion venom (Krämer et al., 2025). Here, we provide a first bioactivity screening for this novel pseudoscorpion venom peptide targeting two insects utilizing injection and feeding assays paired with antimicrobial, hemolytic, and cytotoxicity screenings. Our work provides novel insights to the understudied activity profile and translational potential of pseudoscorpion venom peptides and provides an important basis upon which future arachnid venom bioprospecting programs can be informed upon.

## 2 Materials and methods

### 2.1 Peptide synthesis

The linear venom peptide Ammogarypin has recently been described from *Ammogarypus lawrencei* venom. We outsourced its synthesis via solid-phase synthesis to GenScript Biotech (Rijswijk, the Netherlands). Details on the peptide as provided by the vendor: 1) Molecular weight: 4,682.5 g/mol; 2) Length: 46AA; 3) HPLC purity: 84.2%; and 4) was supplied as a lyophilized powder.

### 2.2 In silico analyses

The 46 amino acid sequence of Ammogarypin was taken from the original work in which it was described (Krämer et al., 2025). The hydrophobicity was evaluated using the heliQuest online analysis tool (Gautier et al., 2008). Molecular weight, Iso-electric point and the net charge at neutral pH were calculated using PepCalc.com (Innovagen AB, Sweden). Peptide structure was predicted using Alphafold3 (Abramson et al., 2024).

### 2.3 Statistical testing of insecticidal activity

To determine whether the survival rates of insects injected with varying venom concentrations differed significantly, we performed one-way fixed-effects ANOVA (Lindman, 2012). We then carried out pairwise comparisons using Tukey’s Honestly Significant Difference (HSD) test (Abdi and Williams, 2010) to identify which specific treatment groups differed significantly. All analyses were performed in the R environment version 4.5.1 (R Core Team, 2025), using the packages car (Fox et al., 2013) and multcomp (Hothorn et al., 2008).

### 2.4 Activity tests in *Myzus persicae*

*Myzus persicae* (Julius-Kühn Institute, Braunschweig, Germany) was reared on healthy 3–4-week-old turnip plants (Brassica rapa L. Tonda A Colletto Viola) in insect rearing tents (BugDorm, MegaView Science, Taiwan) inside a closed, ventilated climate chamber at constant 20 °C with a 16:8 photoperiod and relative humidity of 60%–70%. Plants were replaced regularly. The experiments were conducted using 7-day old nymphs.

For injection assays, nymphs were immobilized using a membrane pump (ILMVAC GmbH, Germany) on a custom-fabricated device constructed from a 200 µL pipette tip (Eppendorf, Germany) and Parafilm (Bemis Company, WI, United States). Exactly 15 nL was administered via microinjection at a rate of 50 nL/s dorsoventrally between the third pair of legs. Following the injection of 20 aphids per treatment, they were transferred in pools of five to Petri dishes containing 2% agar medium and a fresh potato leaf. Mortality was monitored at 1, 3, and 24 h post-injection. Three biological repetitions were conducted.

Feeding assays were carried out using a previously established *in vivo* assay in which a Parafilm mimics the outer leaf or stem sheets including cuticle and epidermis, and the artificial diet the phloem sap (Jancovich et al., 1997; Kirfel et al., 2020). Assays were conducted on a 24-well plate (Sarstedt AG, Germany) that was filled with small glass vials (Agilent Technologies, CA, United States) closing off the wells completely. The peptide was dissolved in nuclease-free water to achieve final concentrations of 500, 250, and 50 ng/μL in the artificial diet respectively (Jancovich et al., 1997). The vials were sealed with two layers of Parafilm (Bemis Company, Inc., WIS, United States) surrounding 20 µL of artificial diet containing yeast extract and sucrose plus 10 µL of diluted peptide. Artificial diet mixed with nuclease-free water and Imidacloprid (4 ppm) were used as controls. The aphids were monitored daily for 4 days. Raw data for all assays performed on *M. persicae* are available as Supplementary Table S1.

### 2.5 Activity tests in *Drosophila suzukii*

*Drosophila suzukii* were tested for insecticidal effects caused by the analyzed toxin as described previously (Peng et al., 2025; Fischer et al., 2024). Briefly, flies were maintained in a ventilated climate chamber at constant 26 °C with a 12:12 photoperiod and a relative humidity of 65% on a diet prepared as described previously (Abdelhafiz et al., 2025). The experiments were carried out using synchronized 3–7-day old flies post-enclosure.

Injection assays were carried out using flies that were anesthetized using CO2 and sorted by sex. We injected in three biological repetitions containing 20 females each. The solubilized peptide with concentrations of 500 ng/μL, 250 ng/μL, 100 ng/μL and 50 ng/μL were injected (46 nL vol) using a free-hand nanoinjector (Drummond Scientific, Broomall, PA, United States). Tap-water and Spinosad (25 ppm) served as negative and positive controls, respectively. Mortality was monitored at 1, 3 and 24 h post-injection.

For feeding assays, 20 flies were starved for 6 h before being transferred to a 50 mL *Drosophila* vial containing twelve 3 µL drops of treatment solution with a concentration of 500 ng/μL on a piece of parafilm. After 6 h and visual confirmation of consumption, the flies were moved to a new vial containing the previously described diet and monitored daily for 4 days. Three biological repetitions were conducted. Raw data for all assays performed on *D. suzukii* are available as Supplementary Video S1, videos of effects following injection of Ammogarypin are presented in Supplementary Table S3.

### 2.6 Antibacterial assay

For antibacterial activity, we carried out assays as disclosed earlier (Hurka et al., 2022). Single colonies from *Staphylococcus aureus* DSMZ 2569 and *Escherichia coli* DSMZ 102053 were picked, transferred to a 15 mL cultivation tube containing 5 mL Tryptic Soy Broth (TSB) media and grown in an incubator for 24 h at 180 rpm and 37 °C. Around 2 mL of the overnight culture was transferred into a new cultivation tube containing 5 mL TSB media and grown for 3–4 h at 180 rpm and 37 °C. After incubation OD_600_ was measured using a BioTek Eon microplate reader and the strains were diluted to 0.00125 for *S. aureus* and 0.000312 for *E. coli*. We seed 96-multiwell plates, in triplicate, exposing the bacteria to 200 μmol/L peptide in 100 µL medium, followed by OD_600_ measurements every 20 min for 48 h following peptide exposure. The growth was normalized to the control cultures in DMSO, and the blank medium. Raw data of the antibacterial activity assay are presented in Supplementary Table S4.

### 2.7 Cytotoxicity assay

Cytotoxicity was determined as previously described (Krämer et al., 2022). Peptide and ionomycin (Cayman Chemical, Ann Arbor, MI, United States) were dissolved in DMSO to create 10 mM stock solutions. MDCK II (Madin-Darby canine kidney) cells were seeded and cultured to 90% confluence in 96-well plates and treated with the compounds (at 100 µM) or DMSO for 48 h at 37 °C in a 5% CO2 atmosphere. Cell viability was determined using CellTiterGlo Luminescent Cell Viability Assay (Promega, Walldorf, Germany). Luminescence was measured in black 96-well plates in a Synergy H4 microplate reader (Biotek, Waldbronn, Germany). Relative light units (RLU) were normalized to the DMSO control set at 100%. Duplicate measurements were used to calculate the means and standard deviations. Raw data of the cytotoxicity assay are presented in Supplementary Table S5.

### 2.8 Hemolytic activity

Hemolytic activity was assessed as described previously (Avella et al., 2024). Briefly, horse blood erythrocytes were purified by adding 900 µL DPBS (Dubelco’s Phosphate-Buffered Saline) to 100 µL of defibrinated horse blood (Thermo Fisher Scientific, Waltham, MA, United States). The cells were centrifuged for 5 min at 804 rcf and 4 °C, the supernatant was discarded and the pellet was resuspended in 1 mL of DPBS. We repeated this process 3 times until the supernatant was clear. A 1% (w/v) erythrocyte suspension in DPBS was used for further analysis. We mixed 50 µL of a 400 μmol/L solution of Ammogarypin in a 96-multiwell v-plate and incubated for 2 h at 37 °C and 130 rpm. Afterwards, the plate was centrifuged for 5 min at 804 rcf and 4 °C, and 50 µL supernatant was transferred into a new plate. We measured the hemolysis photometrically via OD_405_ (Optical Density at 405 nm) in a BioTek Eon microplate reader (BioTek, Winooski, VT, United States of America). A 1% (v/v) Triton X-100 solution served as positive controls, *Apis mellifera* crude venom (50 μg/μL) served as biological controls and DMSO was used as negative control. All measurements were carried out in triplicates. Raw data of the hemolytic assay are presented in Supplementary Table S6.

## 3 Results

### 3.1 Predicted structural and physicochemical properties of Ammogarypin

**FIGURE 1:**
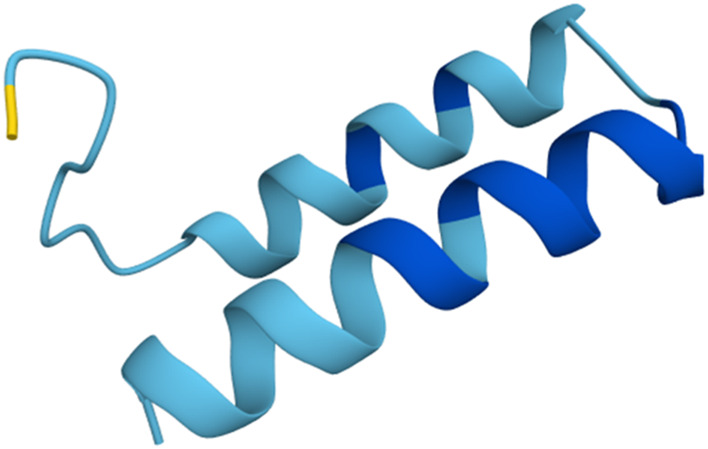
Rendition of Ammogarypin as a double alpha helical molecule. Colors indicate the measure of confidence (pIDDT): dark blue PIDDT >90; light blue 90 > pIDDT >70; yellow 70 > pIDDT >50. Generated using AlphaFold (Abramson et al., 2024).

As a first step towards the functional assessment of Ammogarypin, we employed *in silico* approaches to predict its physicochemical properties and structure. We used the primary structure of the mature peptide (SPVADPEAGILDTIKNVIGKVKGVITDPKVLDAVKAAIAAIKDSLK-CONH2) and subjected it to various tools in order to predict its properties. Our analysis revealed, that Ammogarypin has a hydrophobicity of 35% across its entire sequence and features a molecular weight of 4,682.5 g/mol. At neutral pH, it is a cationic peptide with a net charge of +2 and a calculated iso-electric point (pI) of 9.95. To further gather insights into its structure, we employed AI-based structural predictions via Alphafold3. This revealed that Ammogarypin is indeed a linear peptide that contains two adjacent amphipathic alpha-helical domains (Figure 1).

### 3.2 Ammogarypin is toxic to flies when injected but not when fed

Under natural conditions, pseudoscorpions employ their venoms to facilitate the capture of prey which *inter alia* comprises small insects (Krämer et al., 2019). Therefore, to unveil the biological role of Ammogarypin, we first set out to test its activity on insect models. We selected injection assays into the dipteran *Drosophila suzukii* and the aphid *Myzus persicae* as primary assays to determine the insecticidal activity of Ammogarypin as this application is the best approximation to the natural deployment of pseudoscorpion toxins. Following injection, insects were monitored and regularly checked for a total of 24 h post-injection to capture immediate and long-term effects caused by the peptide.

**FIGURE 2:**
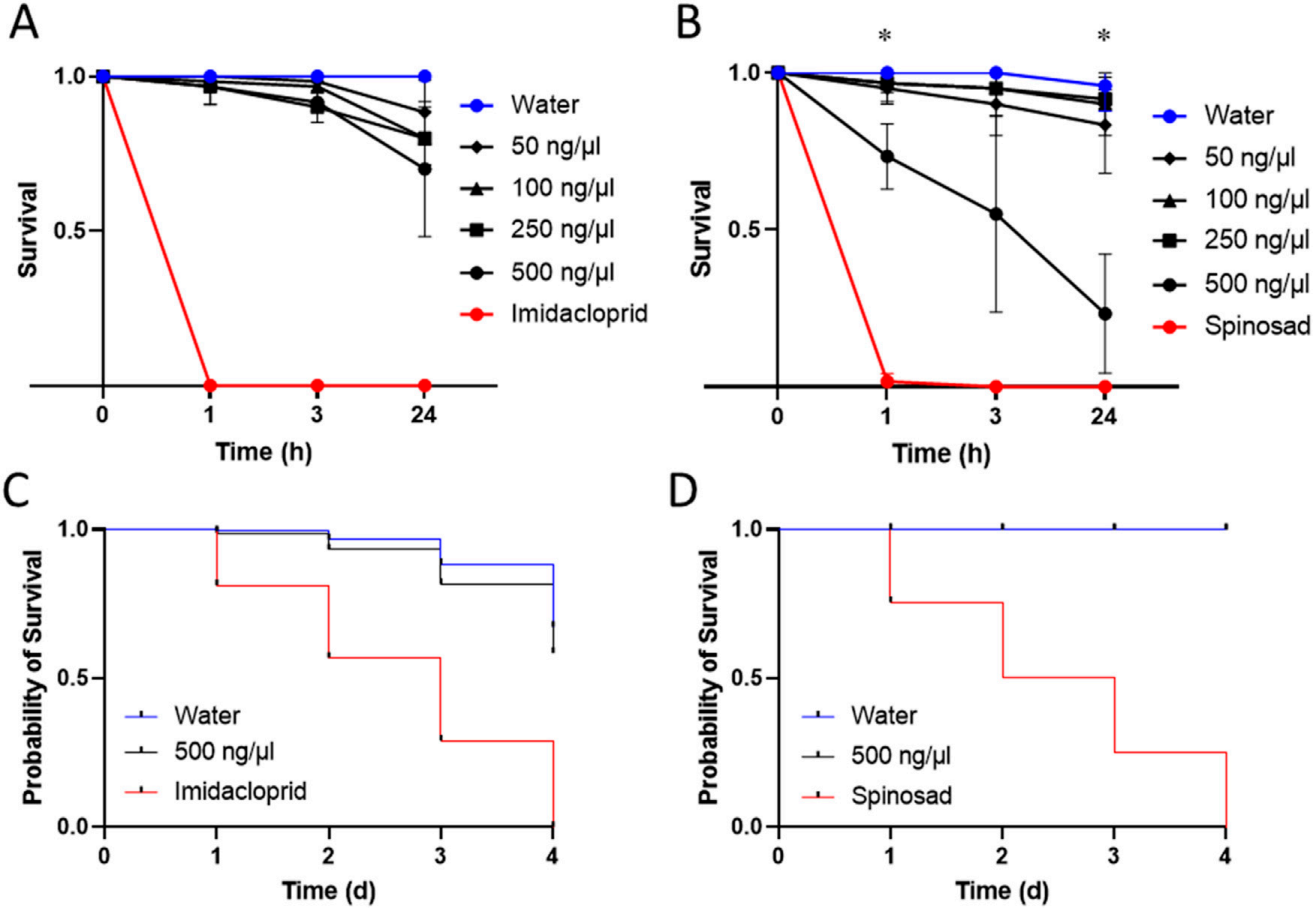
Insecticidal activities of Ammogarypin showing no significant effect on mortality after injection at different concentrations in *M. persicae* **(A)** and resulting in a major increase in mortality in *D. suzukii* at high concentrations of 500 ng/μL **(B)**. Insecticidal activities of Ammogarypin resulting in no effect after oral application in *M. persicae* **(C)** and *D. suzukii* **(D)**. The asterisk (*) indicates statistical significance based on one-way fixed-effects ANOVA testing.

In *D. suzukii*, we observed insecticidal activities when the peptide was administered in higher amounts. Concentrations of 500 ng/μL caused death in 27% (±8%) of flies 1 h post injection, 45% (±25%) after 3 h and 77% (±15%) after 24 h respectively (Figure 2). Lower concentrations caused little to no effects at 1 h and 3 h post injection. Only after 24 h, some marginal insecticidal activity was observed, e.g., at 50 ng/μL exhibiting 17% fatalities (±12%) (Figure 2). Besides counting living and dead insects, we monitored for effects on the insect movement to gather further clues on the function and mode of action of the peptides. Interestingly, across the tested concentrations flies exhibited signs of spastic paralysis when observed at 1 h and 3 h post injection (see Supplementary Table S3), yet in many cases recovered without succumbing to the toxic effects. Therefore, Ammogarypin appears to cause neurotoxic and sometimes fatal effects in Diptera. Next, we injected our peptide into *M. persicae* aphids. This experiment revealed no activity when examined after 1 h and 3 h, yet low insecticidal activity was detected after 24 h at various concentrations (Figure 2). For instance, concentrations of 500 ng/μL and 250 ng/μL caused death in 30% (±18%) and 20% (±7%) of injected aphids respectively (Figure 2). Hence, our data supports that Ammogarypin also has no detrimental effect on aphids, yet to a much lesser extent compared to the dipteran *D. suzukii*.

Previous works have shown that some linear pseudoscorpion toxins cause insecticidal effects when applied orally, thereby offering translational potential as insecticide (Krämer et al., 2022). To test whether Ammogarypin shows similar activity, we proceeded to further test it in feeding assays in both insect models. After feeding, *D. suzukii* and *M. persicae* were monitored for 4 days and their survival was compared to conspecifics reared on nuclease-free water or insecticides (Spinosad for *D. suzukii* and Imidacloprid for *M. persicae*) as controls. Interestingly, in *D. suzukii* no effects were observed even when high concentrations were administered (Figure 2). As for *M. persicae*, we likewise recovered only marginal effects and the calculated probability of survival was above 80% after 3 days (Figure 2). Overall, this suggests, that Ammogarypin exerts much lesser toxicity via oral uptake and, compared to known orally active pseudoscorpion toxins, these effects are much less pronounced.

The one-way fixed-effects ANOVAs revealed a significant effect of peptide concentration on fruit fly survival at 1 h post-injection (F3,8 = 4.98, p = 0.031) and at 24 h post-injection (F3,8 = 9.25, p = 0.005), but not at 3 h post-injection (F3,8 = 3.82, p = 0.057). Specifically, Tukey’s HSD test indicated a significant difference between the 500 and 100 ng/μL concentrations at 1 h post-injection (p = 0.036), and between the 500 ng/μL concentration and all other venom concentrations at 24 h post-injection (250 ng/μL: p = 0.01; 100 ng/μL: p = 0.009; 50 ng/μL: p = 0.016). Meanwhile, the one-way fixed-effects ANOVAs revealed no significant effect of peptide concentration on aphid survival at all measured time points. Details of the results of the performed statistical tests are provided in Supplementary Tables S7, S8.

### 3.3 Ammogarypin has little effects on bacterial growth

**FIGURE 3:**
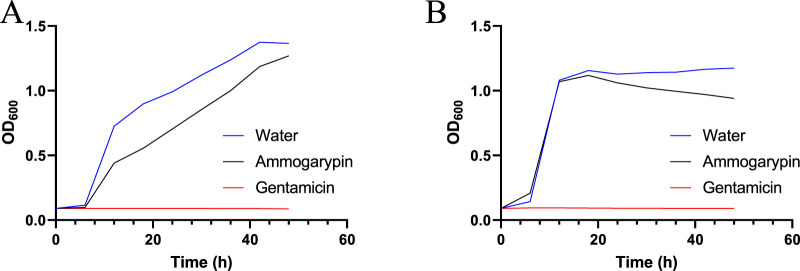
Growth curve of *E. coli* **(A)** and *S. aureus* **(B)** in the presence of 200 μmol/L Ammogarypin over a period of 48 h, measured in 20 min intervals.

In the past it has been shown, that some linear pseudoscorpion toxins exert potent antimicrobial effects against prokaryotes (Krämer et al., 2022; Erkoc et al., 2024). Therefore, antimicrobial screening is an important element in the bioactivity profiling of these peptides. In response, we carried out exploratory screenings to test the toxins effects against two selected bacterial strains, the gram-negative *E. coli* DSMZ 102053 and gram-positive *S. aureus* DSMZ 2569. Both bacterial strains were cultured in a medium containing Ammogarypin at 200 μmol/L and their growth was recorded photometrically using the OD_600_ method. The peptide exhibited a weak effect on the growth of gram-negative *E. coli* (Figure 3A), and no observable effect on gram-positive *S.* aureus (Figure 3B). However, in contrast to other linear pseudoscorpion venom peptides, their activity seems to be of much lesser potency.

### 3.4 No effects on mammalian cells by Ammogarypin

**FIGURE 4:**
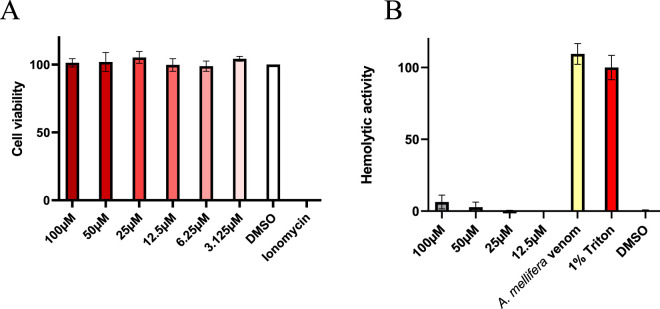
**(A)** Cell viability assay in MDCK II cells showing no effect at tested Ammogarypin concentrations. **(B)** Hemolytic activity of Ammogarypin resulting in no effects against this cell type.

Linear pseudoscorpion venom peptides potentially exert their activities via interaction with lipid bilayers of biomembranes (Erkoc et al., 2024; Pukala et al., 2007; Jenssen et al., 2006). Typical consequences are cytotoxicity and/or hemolytic activity by disruption of membranes and can ultimately lead to cell death (Erkoc et al., 2024; Krämer et al., 2022; Pukala et al., 2007; Dersch et al., 2024). On one hand, these effects could be biologically relevant, e.g., by allowing the peptides to serve as spreading factors or defensive components. However, understanding these aspects is equally important from a translational perspective. Both represent pivotal and unwanted side effects to be explored in preclinical screening of venom peptides and previously other linear pseudoscorpion venom peptides were already shown to cause cytotoxicity and hemolysis. Hence, we next set out to study effects on mammalian cells caused by Ammogarypin. In order to assess the cytotoxic activity of Ammogarypin, a photometric CellTiterGlo Luminescent Cell Viability Assay was conducted on mammalian MDCKII cells (Figure 4A). Interestingly, at neither of the tested concentrations the peptide caused cytotoxicity. To further evaluate the peptides’ capability to damage mammalian cells, we tested it against equine erythrocytes. Reminiscent to our cytotoxicity screening, we did not observe effects against this cell type (Figure 4B). Therefore our *in vitro* assessment targeting cellular structures suggests that Ammogarypin does not exhibit lytic activity on vertebrate cells. With that, it differs profoundly from many other linear pseudoscorpion venom peptides.

## 4 Discussion

Venoms are highly complex chemical cocktails containing diverse pharmacopeias (Herzig, 2019). Whilst several venom derived toxins have already been screened for their potential use in the bioeconomy, particularly as drugs, much remains to be learned about their bioactivities (Trim et al., 2021; Smallwood and Clark, 2021). Recently, the venom systems of arachnids gained particular interest, due to their biological role in facilitating the capture of insect prey (Lüddecke et al., 2022). As this offers the potential to exploit them as novel eco-friendly insecticides, they may hold the potential to generate novel biologic agents for an economic application of pivotal financial- and environmental importance (Windley et al., 2012; Smith et al., 2013). That said, only a miniscule fraction of arachnid toxins have been screened in more detail and especially little is the knowledge about the toxins from the smallest group of venomous arachnids: the pseudoscorpions (Krämer et al., 2025). Only recently, the targeted study of the smallest venomous organisms became possible in an efficient manner thanks to advances in analytical chemistry, sequencing and biotechnology (i.e., Modern venomics) (Reumont et al., 2022). Yet, only few pseudoscorpion venom profiles have been revealed so far and an even smaller number of toxins has been studied to date (Krämer et al., 2019; Krämer et al., 2021; Lebenzon et al., 2021; Santibáñez-López et al., 2018).

In the current work, we employ a broad bioactivity profiling workflow to facilitate the first functional screening of Ammogarypin. This unusually large linear peptide has recently been identified in the venoms of *Ammogarypus lawrencei* and is pending functional characterization (Krämer et al., 2025). We selected various *in vitro* and *in vivo* assays targeting known functionalities of interest which have previously been shown to often play a role for linear pseudoscorpion toxins. Our analysis showed, that Ammogarypin exerts insecticidal activity, likely via neurotoxic mode of action, in flies but not aphids. The results of the performed one-way fixed-effects ANOVA indicate that survival of *Drosophila* significantly differs across the tested concentrations at 1 h and 24 h post-injection. When applied orally, Ammogarypin differed from other previously studied linear pseudoscorpion toxins by not causing noteworthy effects. It further differed tremendously from other related toxins by causing neglectable antimicrobial effects and no activity on mammalian cells. With that our study broadens the current understanding of pseudoscorpion biology and the areas of bioeconomic application of their toxins.

Initially, by facilitating the bioactivity profiling of Ammogarypin, we attempted to understand the translational potential of a novel structurally aberrant linear venom peptide from a pseudoscorpion, particularly in light of potential agricultural exploitation. At least in *D. suzukii*, we observed signs of sporadic paralysis that are consistent with neurotoxic activities that may lead to death. Yet, agricultural utilization is intrinsically bound to oral activity. In this regard, neither in *D. suzukii* nor in *M. persicae*, our data was reflective of activity levels desired for potential agricultural utilization. On one hand, linear arachnid peptides can easily be bioengineered towards increased activity levels, but considering the low starting toxicities of Ammogarypin, it appears strategically more efficient to explore toxins with more distinct effects instead (Dersch et al., 2024). One factor potentially limiting the toxicity of Ammogarypin could be the low antimicrobial effects detected. While past activity screens from linear pseudoscorpion toxins identified highly potent anti-aphid activities in Checacin-family toxins (Krämer et al., 2022), the potency of these peptides has been *inter alia* explained by a mode of action targeting vital symbionts within the aphid gut microbiome (Luna-Ramirez et al., 2017). Owed to the comparatively strongly reduced antimicrobial activity, Ammogarypin seems to be unsuitable to cause similar effects and hence will likely not cause effects with similar impact. Based on this the potential of Ammogarypin for agricultural utilization is limited.

However, our functional screening further allows us to shed more light on the biological role of the tested peptide. The insecticidal activity data indicates, that Ammogarypin features a trophic weapon that is used to overpower insect prey. When tested in Diptera, first fatalities were recorded 1 h post injection. Further, at 1 h we recovered paralytic effects in the flies suggestive of rapid onset of immobilizing neurotoxicity, a major symptom of predatory arachnid toxins (Herzig, 2019; Windley et al., 2012). We observed no significant effects when injecting in aphids, which is suggestive of potential phyletic selectivity which is often encountered in arachnid neurotoxins (Windley et al., 2012; Miyashita et al., 2024). The observed paralyzing effects indicate that Ammogarypin might be a neurotoxin taking effect by binding to ion channels and receptors thereby modulating signal transduction. Though most arachnid neurotoxins are characterized by cysteine-stabilized scaffolds (Langenegger et al., 2019) absent in Ammogarypin, this is no precondition for ion channel binding and for instance several linear peptide toxins with neurotoxic properties were discovered in the venom of scorpions (Ortiz and Possani, 2018; Hancock et al., 1995). It would be a fascinating follow-up work to study the precise mode of action of Ammogarypin using state of the art patch clamp systems, to unveil its electrophysiological profile, and to identify its molecular target. Further, it would be interesting to study Ammogarypin in a broader range of insect systems, particularly Hymenoptera. *Ammogarypus lawrencei*, from which the peptide was identified, is known to be a hunter of ants and, accordingly, bioassays in ants or probing ant receptors would be especially interesting (Krämer et al., 2025).

While the injection data stringently supports a role in prey capture, the remainder of bioassays rules out some of the other dominantly discussed biological functions for linear pseudoscorpion peptides. For instance, the absence of cytotoxicity and hemolytic activity indicates that a function as spreading factor, a component that supports spread of co-administered toxins via disintegration of tissue integrity, is unlikely (Kuhn-Nentwig et al., 2019). Also, a defensive role via the release of cellular contents causing inflammation and nociception, as proposed for other linear venom peptides, appears to be unlikely (Kuhn-Nentwig, 2003; King and Hardy, 2013). Lastly, several linear pseudoscorpion peptides are proposed to be involved in the innate immune system defending the venom gland against microbial colonization (Shafee et al., 2017). Albeit Ammogarypin causes a very marginal growth reductive effect in *E. coli*, the detected reduction is unlikely to be relevant. Therefore, Ammogarypin seems to be a unifunctional linear venom peptide solely used to aid in overpowering insects. This sets it apart from other linear toxins detected in pseudoscorpions which are often multi-pronged and tend to serve multiple biological functions simultaneously. This has important repercussions, as it shows that a large array of bioassays could theoretically be needed to unveil the biological function of a novel peptide and there is no general screening pipeline that efficiently covers all potential avenues of activity venom derived linear peptides may yield. This is especially important, yet counterintuitive, when considering the simple linear nature of these peptides. While these simple folds often give rise to simple and quite unspecific activities (Kuhn-Nentwig, 2003), apparently some more complex and specialised forms emerged in pseudoscorpion venoms. A much deeper functional screening of Ammogarypin and other pseudoscorpion venom peptides is needed to fully grasp their biological significance and functional space.

## 5 Conclusion

Pseudoscorpions belong to the smallest and least investigated venomous animals on earth. Some of their venom components received considerable translational consideration in the last years, but overall, little is known about their function and bioeconomic potential. Our current study sheds light on the biological aspects of Ammogarypin, revealing its role as a trophic element utilized for prey capture. While, from a translational perspective, the toxin has limited potential, our analysis adds important novel data on the functional space occupied by linear pseudoscorpion toxins and will facilitate a more efficient venom biodiscovery from upcoming pseudoscorpion venom profiles and aid in the functional assessment of therein identified biomolecules.

## Data Availability

The original contributions presented in the study are included in the article/Supplementary Material, further inquiries can be directed to the corresponding authors.
